# A Case Report on an Adult Presentation of Henoch-Schönlein Purpura

**DOI:** 10.7759/cureus.26385

**Published:** 2022-06-28

**Authors:** Raja Sood, Priya Parekh, Nitish Raj, Iqra Saani

**Affiliations:** 1 Medicine, New Cross Hospital, Wolverhampton, GBR; 2 Otolaryngology, New Cross Hospital, Wolverhampton, GBR; 3 General Surgery, Medway NHS Foundation Trust, Kent, GBR; 4 Internal Medicine, Medway NHS Foundation Trust, Kent, GBR

**Keywords:** corticosteroids, atypical presentation, late diagnosis, vasculitis, henoch scholein purpura

## Abstract

Henoch-Schönlein purpura (HSP) is an immunoglobulin A (IgA)-mediated multisystem vasculitis commonly affecting children under 10 years of age. Although diagnostic criteria exist, making a diagnosis is often difficult as this condition can present atypically in adults. We discuss a 22-year-old female with a delayed diagnosis of HSP, resulting in significant anxiety and distress. Our patient’s symptoms improved with analgesia and corticosteroids, which were initiated upon diagnosis and she experienced two mild, self-limiting relapses over two years following symptom resolution. Our case illustrates that an integrated multidisciplinary approach is needed to effectively diagnose, safely manage and monitor patients presenting with HSP. Although self-limiting in nature, HSP has the potential to manifest into life-threatening conditions such as end-stage renal failure, which stresses the importance of early diagnosis and management.

## Introduction

Henoch-Schönlein purpura (HSP) is an immunoglobin A (IgA)-mediated systematic vasculitis affecting the vasculature of several systems including the gastrointestinal tract, renal system, skin, and joints [1]. It is characterized by the presence of a vasculitic purpuric rash, abdominal pain, joint pain, renal injury, pulmonary inflammation, or central nervous system involvement [2]. Around 90% of cases occur in children under the age of 10, with a greater preponderance for males [1,3-5]. Although self-limiting in nature, complications such as gastrointestinal hemorrhage and end-stage renal failure may occur [6]. Immunoglobin A nephritis affects 60% of patients and can vary in severity from the presence of asymptomatic microscopic haematuria with proteinuria to irreversible renal failure requiring renal transplantation [7]. Diagnosis of HSP follows the criteria set by the European League Against Rheumatism (EULAR), Paediatric Rheumatology International Trial Organisation (PRINTO), and Paediatric Rheumatology European Society (PRES) [8,9]. These diagnostic criteria include the necessary presence of a palpable purpuric rash with lower limb predominance, no thrombocytopenia or coagulopathy, and at least one of the following: acute abdominal pain, acute arthritis, or arthralgia, kidney involvement, or biopsy showing leukocytoclastic vasculitis.

## Case presentation

A 22-year-old female presented to her general practitioner with a one-day history of sudden onset right-sided abdominal pain, vomiting, and green-colored loose stool. In addition, the patient complained of an acute onset of mild oligoarthropathy affecting her wrists and knees. The patient had recently returned from India and had been recovering from a fever, unremitting cough, sore throat, and generalized lethargy. Her past medical history included acne vulgaris, polycystic ovarian syndrome, allergic rhinitis, astigmatism, and myopia. She took no regular medications and had no known drug allergies. Her social and family history were unremarkable. On examination, she had abdominal tenderness with no guarding or palpable masses and bowel sounds were present. Bedside examination of other bodily systems showed no signs and her vital observations were in normal ranges. The general practitioner suspected a diagnosis of appendicitis and referred this patient to a local surgical assessment unit. The general surgery practitioners took a further history, re-examined the patient, and carried out an investigation with blood including amylase (Table 1) and ultrasound. The suspected diagnosis of appendicitis was ruled out and the patient was discharged with a high dose of codeine/paracetamol for analgesia. 

**Table 1:** Laboratory results CRP: C-reactive protein, eGFR: Estimated glomerular filtration rate

Test	Result	Normal Ranges
Hemoglobin	136 g/L	115-165 g/L
Amylase	55 IU/L	28-100 IU/L
CRP	34 mg/L	0-4 mg/L
Total White cells	5.46 x10^9^/L	4-11 x10^9^/L
Bilirubin	6 mmol/L	<21 mmol/L
eGFR	>90 ml/min/1.73m^2^	>90 ml/min/1.73m^2^

**Figure 1:**
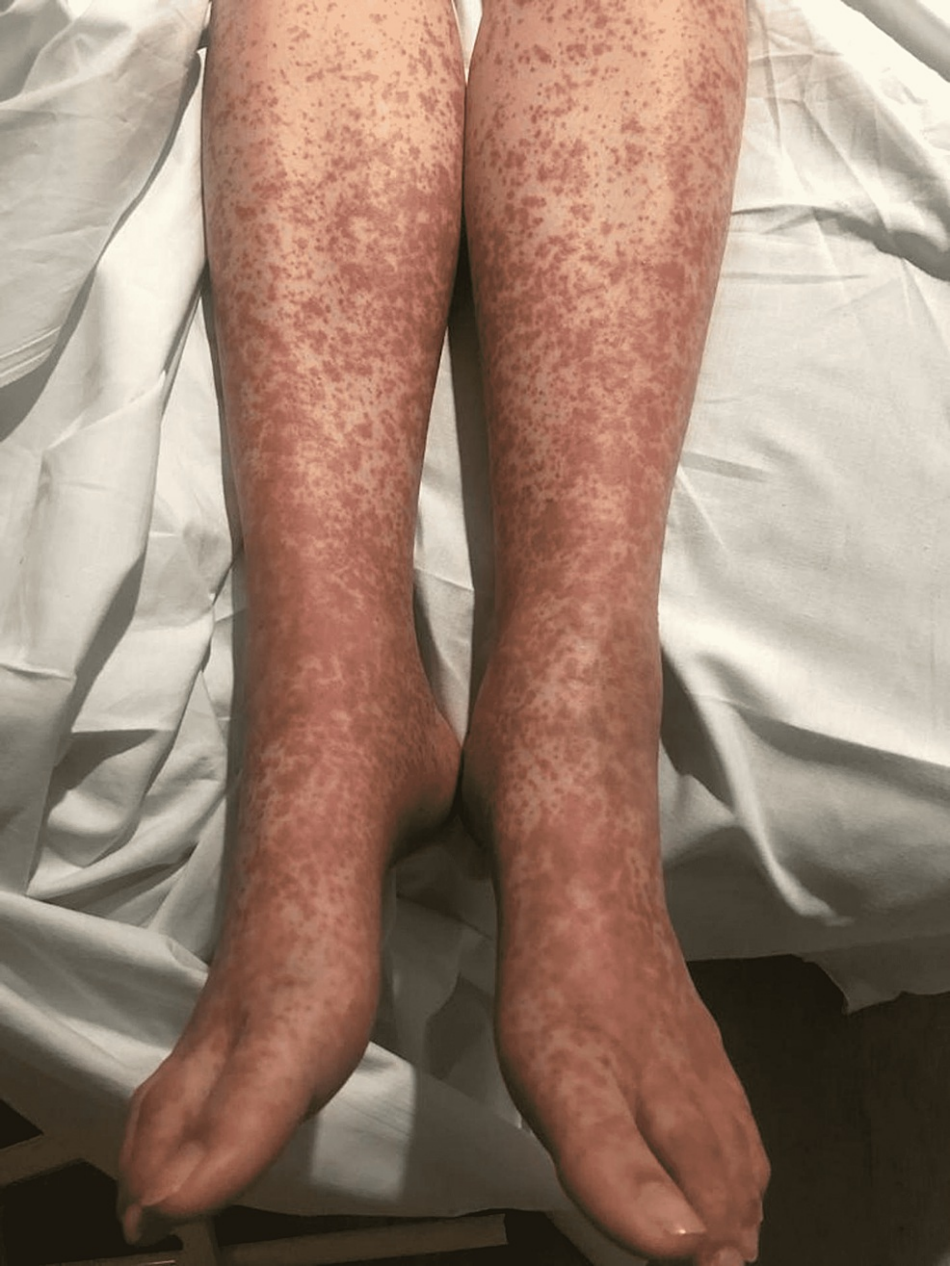
Vasculitic rash

The patient’s pain worsened over the next week, which resulted in recurrent visits to the emergency department where no further definitive diagnosis was made. On the fourth visit to the emergency department, her mid-stream urinalysis was positive for nitrates (+) and protein (++), and microscopy and culture investigations isolated beta-hemolytic group B Streptococci. She was treated for a urinary tract infection with nitrofurantoin. Six days later, she represented to the emergency department after developing a purpuric vasculitic rash on her buttock (Figure 1), lower limbs, and upper limbs and worsening oligoarthropathy. The laboratory blood findings showed a neutrophilic leucocytosis with a white cell count of 16.3 109/L and inflammatory markers were raised (C-reactive protein (CRP) 54 mg/L and erythrocyte sedimentation rate 39 mm/h). The rest of the full blood count, glomerular filtration rate, urea and electrolytes, liver function tests, coagulation panel, and cardiac enzymes were normal. A repeat urine sample was positive for blood (+++), and protein (++), and the albumin-to-creatinine ratio was raised to 27.2.

The clerking clinicians sought the opinion of the rheumatology team who carried out the following immunological and infectious investigations: blood cultures, urine culture, thiopurine S-methyltransferase, human immunodeficiency virus, hepatitis serology, chlamydia, M*ycoplasma pneumonia*, rheumatoid factor (RF), anti-cyclic citrullinated peptide (anti-CCP), antineutrophil cytoplasmic antibodies (ANCA), double-stranded DNA and complement levels, all of which were within normal ranges. The antinuclear antibody (ANA) level was weakly positive with a finely speckled titer of 1:160. Due to ongoing abdominal pain, a repeat abdominal ultrasound showed excess fluid within the small bowel and no other organ pathology. Computed tomography of the abdomen and pelvis showed mild thickening of the duodenal wall with an increased enhancement of the distal duodenum and jejunum, suggesting mild inflammatory changes consistent with probable infective enteritis. No signs of Crohn’s disease or appendicitis were seen on imaging. A dermatologist reviewed the patient and performed a skin biopsy that showed increased IgA deposition and was confirmatory with an inflammatory leukocytoclastic vasculitic process, and a potent topical corticosteroid was prescribed. Based on these investigation findings and the clinical presentation, a diagnosis of HSP was suspected by the rheumatologist.

Following the multi-disciplinary team meeting involving dermatologists, rheumatologists, immunologists, and other healthcare professionals, the patient was treated for HSP with a course of prednisolone (30 mg for two weeks) which was then weaned down. The decision to start biologics (such as azathioprine) was decided against due to the resolution of symptoms with steroid medication. Over the next two weeks, the patient’s abdominal and joint pains subsided, and the rash improved. However, proteinuria did not improve, and the albumin-to-creatinine ratio remained high. Further opinion from the renal team was sought and they advised the monitoring of the albumin-to-creatine ratio, which normalized over the next two weeks to 3.1 from 27.2. The patient was discharged to the care of the General Practitioner with the advice to monitor urine for proteinuria. A follow-up one year later revealed the patient’s condition was completely resolved and all markers returned to normal. The patient complained of two mild relapses of the rash without other symptomology, which self-resolved within a few days.

## Discussion

Henoch-Schönlein purpura is the most common systemic vasculitis in children with an annual incidence of six to 22 per 100,000 person-years in children and 3.4 to 14.3 per 100,000 person-years in adults [9]. The mean age of onset of HSP is six years old and it is twice as common in males than in females [1,4,5]. There are few reports presenting HSP in female adults as discussed and the atypical demographics of our patient could account for her delayed diagnosis.

Examination plays a key role in diagnosing HSP and there are several common findings. Dermatological manifestations involve a symmetrical non-tender pruritic erythematous, macular, or urticarial rash, which develops into palpable purpura with blanching papules. The distribution of this rash varies depending on the age of the patient. Children under one-year-old develop a widespread rash involving the face, torso, and upper extremities, whilst in toddlers the rash typically involves the lower back and buttocks. In older children and adults, the rash is usually isolated to the lower limbs and buttocks [10]. Our patient’s initial cutaneous symptoms were consistent with the usual course of HSP in adults as described in the literature but progressed in an atypical manner affecting the upper limbs.

Other examination features of HSP include arthralgia and gastrointestinal disturbance. Arthritis and joint pains are more commonly featured in older children and adults. In addition, adult-onset HSP is more likely to present with arthralgia without arthritis [10]. The pattern of joint involvement is typically that of an oligoarthritic picture without redness, warmth, or erythema. In our case, the patient presented with acute-onset pain in three joints with crampy abdominal pain. Despite this typical presentation, we suspect that the lack of cutaneous findings contributed to the missed diagnosis and exploration of a surgical abdomen.

Renal involvement is one of the main indicators of morbidity from HSP. Around 30% to 50% present with haematuria and or proteinuria within six weeks and the likelihood of developing renal pathology increases with the age of onset [11]. This is normally self-limiting, but around 7% develop a long-term nephritic or nephrotic condition and 1% develop end-stage renal failure. Severe renal involvement including progression to nephrotic syndrome and end-stage renal failure is more common in the adult population [12]. Our patient was found to have proteinuria and haematuria on urinalysis with a raised albumin-to-creatinine ratio, which is consistent with HSP nephritis.

Although HSP is a clinical diagnosis, laboratory studies and imaging may help in more atypical cases. As well as routine laboratory studies, an extensive immune panel of blood may be required to support the diagnosis and rule out alternate pathophysiology, including ANA, ANCA, RF, and factors VIII and XIII levels. Imaging can also be considered, such as renal or skin biopsy, which may play a role when the diagnosis is uncertain or in monitoring for possible complications and system involvement.

The management of HSP is largely supportive and involves a combination of analgesia, anti-emetics, hydration, and monitoring for complications. Treatments aim to provide acute symptom relief and prevent renal deterioration. Cutaneous involvement does not usually require management [13]. As HSP is characterized by IgA deposition and white cell infiltration within blood vessel walls, corticosteroids can play a role in inhibiting this inflammatory process [14]. One study found that 1 mg to 2 mg per kg of oral prednisolone for two weeks is effective for abdominal and joint symptoms. Other studies looking into the role of corticosteroids in HSP have found that although steroids do not prevent the onset of renal involvement, they are helpful for symptomatic relief (especially of abdominal and joint pains), as was the case in our patient whose symptoms resolved following steroids [14,15].

Henoch-Schönlein purpura is usually self-limiting. Most patients completely recover with symptom resolution within eight to 10 weeks of onset and 5% develop chronic symptoms [16]. Complete clinical resolution is more likely in patients with mild renal involvement, no neurological complications, and a disease course of less than six weeks. Disease recurrence may occur in 30% to 50% of patients as late as seven years after the initial onset, and long-term follow-up studies have shown delayed-onset chronic kidney disease as a complication in cases where steroids were used in management [17].

## Conclusions

An integrated multidisciplinary approach is needed to effectively diagnose and safely manage and monitor patients presenting with HSP. Although self-limiting in nature, HSP has the potential to manifest into life-threatening conditions such as end-stage renal failure. This emphasizes the importance of early diagnosis and management. Our case sets a precedence for clinicians to consider HSP in a wider demographic profile who may present atypically.
